# Nanoscale flow cytometry reveals interpatient variability in HIV protease activity that correlates with viral infectivity and identifies drug-resistant viruses

**DOI:** 10.1038/s41598-020-75118-1

**Published:** 2020-10-22

**Authors:** Michał M. Bonar, Caroline O. Tabler, Aiman A. Haqqani, Lauren E. Lapointe, Jake A. Galiatsos, Samira Joussef-Piña, Miguel E. Quiñones-Mateu, John C. Tilton

**Affiliations:** 1 Center for Proteomics and Bioinformatics, Department of Nutrition, School of Medicine, Case Western Reserve University, Cleveland, OH 44106 USA; 2 Department of Pathology, School of Medicine, Case Western Reserve University, Cleveland, OH 44106 USA; 3 Present Address: Department of Microbiology and Immunology, University of Otago, Dunedin, 9016 New Zealand

**Keywords:** Proteases, Microbiology, Retrovirus, Flow cytometry

## Abstract

HIV encodes an aspartyl protease that is activated during, or shortly after, budding of viral particles from the surface of infected cells. Protease-mediated cleavage of viral polyproteins is essential to generating infectious viruses, a process known as ‘maturation’ that is the target of FDA-approved antiretroviral drugs. Most assays to monitor protease activity rely on bulk analysis of millions of viruses and obscure potential heterogeneity of protease activation within individual particles. In this study we used nanoscale flow cytometry in conjunction with an engineered FRET reporter called VIral ProteasE Reporter (VIPER) to investigate heterogeneity of protease activation in individual, patient-derived viruses. We demonstrate previously unappreciated interpatient variation in HIV protease processing efficiency that impacts viral infectivity. Additionally, monitoring of protease activity in individual virions distinguishes between drug sensitivity or resistance to protease inhibitors in patient-derived samples. These findings demonstrate the feasibility of monitoring enzymatic processes using nanoscale flow cytometry and highlight the potential of this technology for translational clinical discovery, not only for viruses but also other submicron particles including exosomes, microvesicles, and bacteria.

## Introduction

HIV-1 encodes an aspartyl protease (PR) that is encoded as a monomer within the viral Gag-Pol polyprotein but functions as a mature dimer. During or shortly after virus budding, PR dimerizes, undergoes autocatalytic processing from the polyprotein, and subsequently cleaves the viral Gag and Gag-Pol polyproteins into individual subunits in a highly ordered process called maturation. This PR-dependent maturation is characterized by the rearrangement of the Gag capsid proteins into an elongated conical shell and condensation of the Gag nucleocapsid proteins that stabilize the dimeric viral RNA genome. Viruses that fail to undergo PR-dependent maturation are noninfectious^1–5^ and PR inhibitors are a major class of antiretrovirals used in the treatment of HIV-infected patients (reviewed in^6–8^). Accurate monitoring of PR activity is critical to evaluate novel drugs and viral resistance, host and viral factors that regulate PR dimerization and activation, and patient-to-patient heterogeneity that could impact disease progression.

A variety of techniques to monitor HIV PR function are available including cell-free assays with recombinant PR, western blot analysis of Gag and Gag-Pol polyprotein processing, and electron microscopy. Cell-free assays are robust and high throughput but do not mimic the physiological conditions of viral assembly and release. Moreover, since many use recombinant PR, the effects of other viral and host factors on PR activation cannot be investigated. Western blot analysis of PR activity is relatively straightforward, but quantification relies on densitometry (band intensity) that is frequently non-linear^9,10^ and therefore can be inaccurate. Western blots also provide aggregate data on large numbers of viruses, obscuring potential viral heterogeneity in PR activation. Electron microscopy can analyze maturation within single viruses but is low-throughput, labor intensive, comparatively expensive, and is limited in other parameters that can be monitored. An attractive alternative is the emerging field of *nanoscale* flow cytometry (NFC), which promises high-throughput, multiparametric characterization of individual viruses as well as other submicron particles including exosomes, microvesicles, mitochondria, and bacteria.

NFC evaluation of viruses, sometimes called ‘flow virometry’, has revealed viral heterogeneity in host protein incorporation^11^, levels and conformations of viral proteins^12–14^, size^12,15^ and genome incorporation^16^. Importantly, several studies have demonstrated the ability to sort viruses while maintaining their infectivity^8,10,12–14^, enabling downstream functional analysis of viral subpopulations to investigate how heterogeneity impacts viral fitness, transmission, and immunity. However, to date, NFC has been limited to monitoring protein levels and conformations and has not been applied to monitoring enzymatic processes such as HIV PR activity.

In this study, we developed a sensitive NFC-based FRET reporter that undergoes colorimetric changes in individual viral particles in the presence of functional HIV PR, named VIPER for VIral ProteasE Reporter. Processing of VIPER was highly reproducible and correlated strongly with processing of the Gag polyprotein, demonstrating that it reliably detects PR activity. Processing occurred in the presence of mutations that block full maturation of the PR dimer through autocatalytic processing, indicating that VIPER detects precursor PR dimerization and activation, an understudied step of maturation. NFC detection of HIV virions is also highly quantitative^17^ and simultaneous measurements of PR activity and virion release in viruses bearing mutations in the HIV *integrase* gene revealed complex phenotypes that led to rapid mechanistic insights. We next studied PR activation in patient-derived viruses and observed substantial interpatient variability in PR activity that correlated with viral infectivity. This heterogeneity in PR activity in patient-derived viruses has not previously been reported and has important clinical implications. Finally, we demonstrate that NFC can distinguish between PR inhibitor-sensitive and -resistant patient-derived viruses, accurately predicting sensitivity of all 20 (100%) antiretroviral naïve or sensitive viruses and 13 of 16 (81%) of PI and challenging multi-PI resistant viruses.

Together, these results represent a significant advancement in monitoring HIV protease activity, enabling sensitive, high-throughput detection of HIV PR activation within individual virions that can be used to monitor precursor PR activation and the interplay between PR activity and viral particle release. To our knowledge, this study represents the first use NFC to investigate an enzymatic process, thereby greatly expanding the potential of this powerful technology for analysis of viruses, extracellular vesicles, and other submicron particles. These experiments also demonstrate that NFC can uncover previously unappreciated heterogeneity in patient-derived viruses and can detect drug-resistant viruses, highlighting the important clinical implications of nanoscale flow cytometry.

## Results

### Generation of a FRET-based reporter (VIPER) for analysis of HIV protease activity in individual viruses

**Figure 1:**
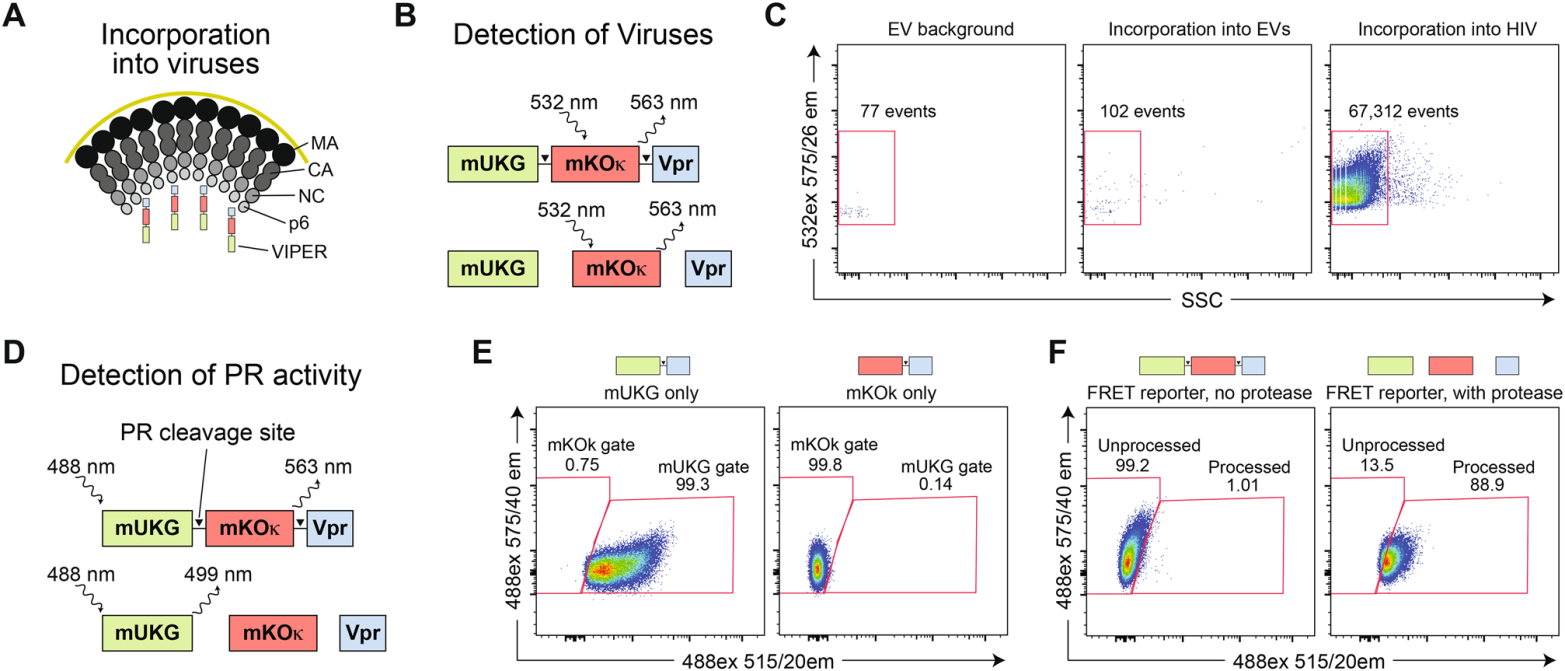
The VIral ProteasE Reporter (VIPER) is incorporated into viral particles and undergoes colorimetric changes in the presence of active HIV protease. (**A**) Schematic of VIPER reporter incorporation into budding particles. The HIV accessory protein Vpr is packaged into viral particles due to a non-covalent interaction with the Gag p6 structural protein. (**B**) Schematic of the VIPER reporter construct and detection of viruses. VIPER consists of a mUKG and mKOκ fluorescent pair separated by an HIV protease (PR) cleavage site and fused to the HIV accessory protein Vpr. Viruses can be identified using direct stimulation of the mKOκ subunit using a 532 nm laser, allowing for detection of viral particles by fluorescence thresholding irrespective of PR processing of the reporter. (**C**) For assessment of specific incorporation of the reporter into viruses, HEK293T cells were transfected with control plasmids (machine noise and EV background), the VIPER construct alone (incorporation into EVs), or the VIPER with HIV (incorporation into HIV). Supernatants were harvested, filtered, and analyzed by nanoscale flow cytometry (NFC). VIPER was robustly and selectively incorporated into HIV particles. (**D**) Schematic of the detection of HIV PR activity. Stimulation with a 488 nm laser results in differential emission depending on whether the viral PR has cleaved the reporter. (**E**) Control constructs containing mUKG-Vpr or mKOκ-Vpr alone were generated and used to set gates for nanoscale flow cytometry (NFC) analysis. (**F**) NFC analysis of viral particles incorporating VIPER demonstrated robust colorimetric changes in the presence of an active HIV protease.

To analyze HIV protease (PR) activity in single viral particles we chose to employ Förster resonance energy transfer (FRET), a technique previously used to monitor PR activity in cell-free and cell-based assays^18–21^. We employed the mUKG-mKOκ fluorescent protein pair based on its good spectral separation, compatibility with a wide range of flow cytometry instruments, and high quantum yield that reflects efficient energy transfer between the fluorescent proteins. The PR cleavage sequence SQNYPIVQ, which separates the matrix (MA) and capsid (CA) subunits of the HIV Gag structural proteins, was inserted between mUKG and mKOκ. This reporter was linked to the viral accessory protein Vpr that makes noncovalent interaction with the Gag p6 protein^22^, which we anticipated would result in specific incorporation of the reporter into viral particles (Fig. 1A). Hereafter, this mUKG-mKOκ-Vpr FRET reporter is called VIPER (for VIral ProteasE Reporter). To detect viral particles, we took advantage of the feature that the mKOκ subunit of VIPER can be directly stimulated by a green (532 nm) laser, resulting in emission at 563 nm irrespective of whether the PR cleavage site has been processed (Fig. 1B). To validate incorporation into HIV virions, we harvested supernatants from cells transfected with control plasmids, VIPER plasmid alone, or plasmids encoding VIPER and a NL4-3 HIV core. Supernatants were diluted to ensure single particle detection and the number of events collected over 20 s recorded (Fig. 1C). As expected, only a small number of events were seen with control supernatants, likely reflecting machine ‘noise’ or autofluorescence from extracellular vesicles (EVs). Importantly, cells transfected with the reporter alone resulted in similar numbers of events, indicating that the reporter is not packaged efficiently into EVs. In contrast, high numbers of fluorescent particles were detected in cells cotransfected with the reporter and HIV, demonstrating specific and robust incorporation of the reporter into viral particles.

To monitor PR activity, the FRET aspect of VIPER was stimulated using a blue (488 nm) laser. In the absence of functional PR, the mUKG and mKOκ subunits should remain connected, resulting in a FRET transfer from mUKG to mKOκ with subsequent emission at 563 nm. In contrast, if PR processes the cleavage site, mUKG and mKOκ are physically separated, abolishing FRET and resulting in emission from mUKG at 499 nm (Fig. 1D). To set gates for NFC detection of processed and unprocessed viruses, we generated mUKG-Vpr and mKOκ-Vpr controls. Both fluorescent proteins can be directly stimulated by the 488 nm laser, although this wavelength is suboptimal for mKOκ and generates only modest fluorescence (Fig. 1E). Next, we generated viruses packaging VIPER with or without functional PR proteins. In the absence of PR, fluorescence from VIPER was almost entirely in the unprocessed (mKOκ) flow cytometry gate, whereas in the presence of functional PR a colorimetric shift in the viruses was observed (Fig. 1F). Collectively, these results demonstrate that VIPER is specifically incorporated into individual virions and undergoes a detectable colorimetric change in the presence of functional HIV PR.

### VIPER is a sensitive and reproducible indicator of HIV PR activity and correlates well with processing of the Gag structural proteins

**Figure 2:**
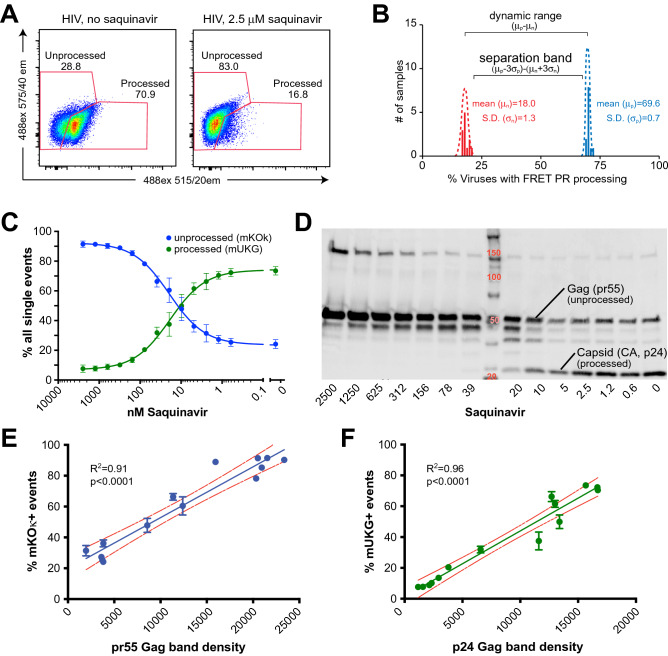
VIPER is a sensitive and reproducible indicator of HIV protease activity and correlates well with processing of the viral structural proteins. (**A**) Representative nanoscale flow cytometry (NFC) plot showing processing of VIPER in the presence or absence of 2.5 μM of the HIV protease inhibitor saquinavir. (**B**) Z-factor (Z′) analysis of 12 independent preparations of HIV generated in the presence or absence of saquinavir. The Z′ is equivalent to the separation band minus the dynamic range of the assay. The Z′ for the VIPER assay was 0.88. (**C**) Increasing concentrations of the protease inhibitor saquinavir inhibit processing of VIPER in a dose-dependent manner. (**D**) Western blot of HIV over a range of saquinavir concentrations. The unprocessed Gag polyprotein (pr55) and processed capsid (CA) subunit (p24) are indicated. (**E**) The percentage of unprocessed (mKOκ) events by NFC correlated well with the concentration of unprocessed Gag (pr55). (**F**) Detection of processed viruses by NFC (mUKG) also correlated well with the concentration of processed p24 CA by western blot.

HIV PR is the target of nine active, FDA-approved protease inhibitors (PIs). To further validate VIPER, we generated viruses using NL4-3—a patient-derived HIV isolate that is widely used in the field—in the presence or absence of high doses of the PI saquinavir. As expected, viruses generated in the presence of saquinavir demonstrated significantly reduced processing of VIPER compared to untreated particles (Fig. 2A). Interestingly, processing of VIPER in viruses exposed to high-dose saquinavir was slightly higher than in the absence of PR, suggesting that some processing occurs even at high drug concentrations. To evaluate the reproducibility of the VIPER assay, we generated 12 independent preparations of NL4-3 HIV with or without saquinavir and performed Z factor (Z′) analysis, a measure of reproducibility used to determine the suitability of an assay for high-throughput screening^23^. The Z′ for VIPER was 0.88, indicating that this assay is highly reproducible (Fig. 2B). Next, we evaluated VIPER processing over a wide range of saquinavir concentrations. As anticipated, we observed a dose-dependent reduction in cleavage of VIPER with increasing levels of saquinavir (Fig. 2C). Concurrently, we analyzed processing of the Gag structural proteins by western blot (Fig. 2D). Unprocessed Gag migrates at a molecular weight of ~ 55 kDa, whereas the processed CA subunit has a molecular weight of 24 kDa and the relative band intensities were calculated using Image-J. The intensity of the unprocessed Gag by western blot correlated very well with the percentage of viruses in the unprocessed mKOκ gate (R^2^ = 0.91, p < 0.0001, Fig. 2E). Similarly, the band intensity of the processed p24 CA by western blot correlated extremely well with viruses in the processed mUKG gate (R^2^ = 0.96, p < 0.0001, Fig. 2F). Together, these results indicate that VIPER is a sensitive and reproducible indicator of HIV PR activity that correlates well with processing of the Gag structural proteins.

### VIPER detects activation of the precursor protease embedded in the Gag-Pol polyprotein

**Figure 3:**
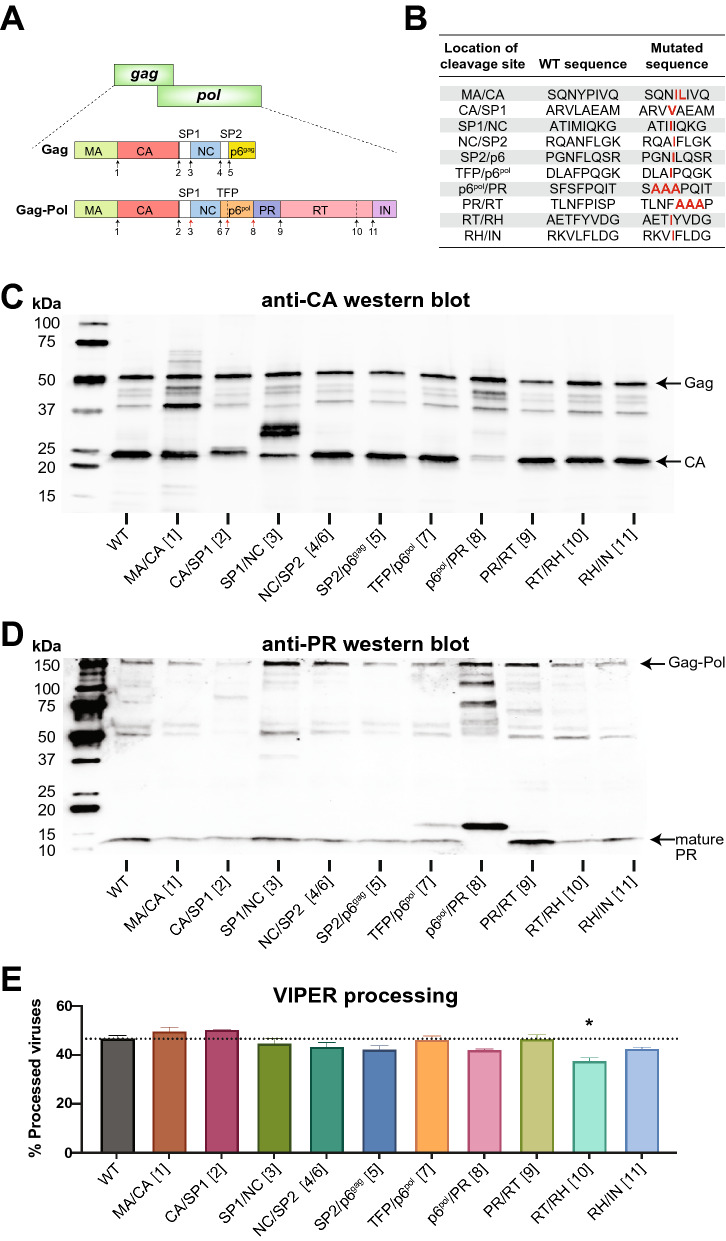
VIPER can be processed by precursor HIV-1 PR that has not undergone full autocatalytic processing. (**A**) Schematic of HIV-1 Gag and Gag-Pol with arrows indicating the 11 canonical PR cleavage sites. Red arrows correspond to cleavage sites processed by the ‘precursor’ PR that has lower stability and processing efficiency compared to the mature PR. Black arrows indicate cleavage sites processed by mature PR. (**B**) Table of mutations in HIV-1 PR cleavage sites reported to block processing. Due to overlap in sequence, mutations at the NC/SP2 processing site (site 4) in Gag also inhibit processing of the NC/TFP junction (site 6) in Gag-Pol. (**C**) Western blot analysis of Gag processing using an anti-p24 antibody that detects CA and its processing intermediates. Multiple cleavage site mutations altered processing, with particularly strong effects noted for mutations of the SP1/NC junction (site 3) and the p6pol/PR junction (site 8) that are processed during the formation of the mature PR. (**D**) Western blot analysis of Gag-Pol processing using an anti-PR antibody that detects the mature, 10.8 kDa protease and its precursors. Most cleavage site mutations did not affect formation of the mature protease, but the p6pol-PR mutation completely abolished formation of the mature PR. (**E**) Processing of HIV-1 virions as detected by NFC using VIPER. Although some of the cleavage site mutations resulted in slight decreases in processing, none reached statistical significance except for processing at the RT/RH junction of reverse transcriptase (site 10, p = 0.023); however, this was not significant after correction for multiple comparisons.

Although HIV-1 PR functions as a mature dimer, only a single monomer is encoded within each Gag-Pol polyprotein. In a process that is still poorly understood—particularly in the physiological context of viral assembly and budding from cells—two PR monomers embedded in Gag-Pol polyproteins dimerize and gain weak catalytic activity. This precursor form of the HIV PR cuts Gag-Pol in an autocatalyic, intramolecular reaction at the SP1-NC, the transframe peptide (TFP)-p6^pol^, and p6^pol^-PR cleavage sites (Fig. 3A), resulting in the formation of a mature, 10.8 kDa PR that cleaves the remaining Gag and Gag-Pol cleavage sites in a highly ordered process.

To determine whether VIPER was processed by the precursor or mature PR, we generated NL4-3 viruses containing mutations within canonical PR cleavage sites in Gag and Gag-Pol that have previously been reported to prevent efficient processing^2,24,25^ (Fig. 3B). We first analyzed the mutant viruses using western blot and observed alterations in Gag processing consistent with the expected impairments in processing (Fig. 3C). For instance, the MA-CA cleavage site mutation resulted in enhanced detection of the 41 kDa MA-CA processing intermediate, while the CA-SP1 mutation resulted in shift of the CA band from 24 to 25 kDa that reflects the continued presence of the small SP1 peptide. Similarly, mutagenesis of the SP2-p6 junction eliminated the 41 kDa band corresponding to Gag with the p6 domain removed. The SP1-NC and p6^pol^-PR cleavage site mutations, which affect sites cleaved by the precursor HIV-1 PR, resulted in significant alterations in Gag processing as expected. In contrast, the TFP-p6^pol^ mutation had no significant effects upon Gag processing by PR. Having characterized the effects of cleavage site mutations on Gag processing, we used western blot to directly assess the formation of the mature, 10.8 kDa viral protease (Fig. 3D). Again, we expected that mutations in SP1-NC, TFP-p6^pol^, and p6^pol^-PR cleavage sites could affect formation of the mature PR since the precursor protease cleaves these sites during autoprocessing and formation of a mature PR^26^. Mutation of the SP1-NC junction, while affecting Gag processing, did not prevent the formation of a mature 10.8 kDa protease. In contrast, mutation of the TFP-p6^pol^ cleavage site resulted in the appearance of a higher molecular weight (~ 15 kDa) protease precursor in addition to the 10.8 kDa mature protease. Finally, mature PR was completely absent in the viruses containing mutations in the p6^pol^-PR cleavage site, indicating that in these viruses the PR is exclusively in an immature, precursor conformation. Next, we evaluated processing of VIPER in the presence of cleavage site mutations using NFC (Fig. 3E). The only cleavage site mutation that resulted in significantly impaired processing of VIPER by NFC analysis was the junction within reverse transcriptase (RT) between the RT polymerase and RNase H (RH) domains that results in the formation of a functional p66/p51 heterodimer (p = 0.023, not significant after correction for multiple comparisons). Importantly, mutagenesis of p6^pol^-PR cleavage site that completely inhibits formation of the mature PR did not significantly reduce processing of VIPER, indicating that the reporter can detect the activity of the precursor PR. These data indicate that VIPER monitors the earliest stages of PR dimerization and autocatalysis within intact viral particles. Of note, while processing of VIPER correlates well with Gag processing with wild-type protease (Fig. 2), certain mutations in Gag and Gag-Pol cleavage sites—such as p6^pol^-PR—can severely impact Gag and Gag-Pol processing while VIPER processing remains unaffected. We believe these discrepancies are explained by differences in the sensitivity of the substrates—Gag, Gag-Pol, or VIPER—to processing by precursor or mature PR.

### Nanoscale flow cytometry analysis of viruses enables rapid detection and classification of mutants by their effects on protease processing and virus release

One of the primary goals in the development of the NFC-based assay was to identify host and viral factors that regulate the formation of the precursor PR in intact viruses, particularly mutations that lie outside of PR gene. To assess this, we generated NL4-3 viruses containing mutations in the viral integrase (IN) protein that have previously been reported to affect the activity of the HIV-1 protease. The D116A and E152E mutations in the catalytic site of IN have been reported to result in altered morphology of virions by electron microscopy, suggesting a defect in PR-mediated Gag polyprotein processing and viral maturation^27^. A third mutation was made in the N-terminal region of IN, H12N, that blocks the ability of IN to form a tetrameric complex^28^ and results in significant disruption of virion morphology by electron microscopy^29^. As controls, we also generated wild-type NL4-3 viruses and viruses with the PR D25N mutation that inactivate the catalytic site of PR.

**Figure 4:**
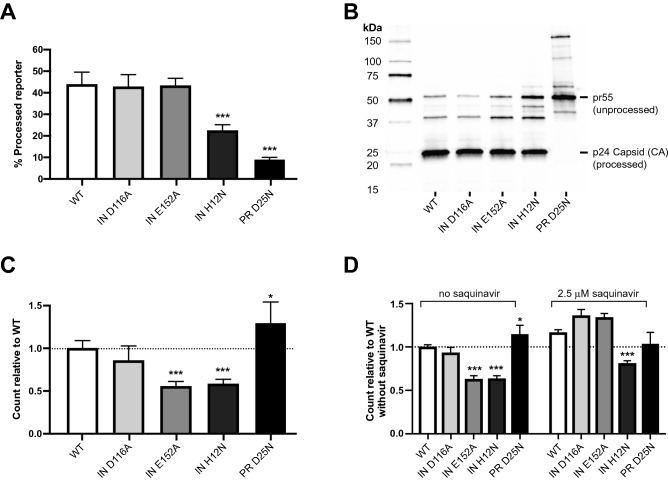
NFC enables rapid detection of mutations affecting PR processing efficiency and virus release. (**A**) PR processing of VIPER with wild-type NL4-3, IN D116A, IN E152A, IN H12N, and PR D25N mutants, analyzed using NFC. The IN H12N and PR D25N mutations both significantly reduced processing efficiency (p < 0.0001 for both). (**B**) Western blot analysis of p24 CA processing with wild-type NL4-3 and mutant viruses. The PR D25N mutant resulted in severe disruption of Gag and Gag-Pol processing as expected for the catalytic site mutation. The IN H12N and E152A mutants resulted in slight elevations in unprocessed Gag and processing intermediates. (**C**) Release of HIV-1 viruses as measured by NFC. The IN E152A and H12N mutations resulted in significant reductions in virus release (p < 0.0001 for both). The PR D25N mutation resulted in a modest but significant increase in virus release (p = 0.021). (**D**) Virus release for the E152A mutant was fully restored by treating cells with saquinavir. However, the H12N mutant continued to demonstrate significantly reduced processing (p < 0.001) in the presence of saquinavir. Virus release in the absence of saquinavir is replotted from the data shown in part (**C**).

NFC analysis revealed that IN D116A and IN E152A mutations did not alter the percentage of virions that processed VIPER, whereas the IN H12N and PR D25N mutations both significantly reduced the percentage of viruses with processed VIPER (Fig. 4A, p < 0.0001 for both). Western blot analysis of these viruses confirmed that the PR D25N mutation results in profound alterations to Gag and Gag-Pol processing; in contrast, IN H12N and IN E152A resulted in relatively minor alterations including modest elevations in unprocessed pr55 Gag and several processing intermediates (Fig. 4B). The IN D116A mutant exhibited processing indistinguishable from wild-type NL4-3.

Next, we took advantage of the ability of NFC to accurately and sensitively count HIV-1 virions^17^ to assess the effects of mutations on viral particle release from cells. Here, a different pattern was observed, with the IN D116A having wild-type levels of virion release, the IN E152A and IN H12N mutants demonstrating significantly impaired particle release (Fig. 4C, p < 0.0001 for both), and the PR D25N mutation resulting in a modest increase in virus release (p = 0.021). Virion release and PR activation are correlated: activation of PR before assembly and budding is complete results in premature processing of Gag and Gag-Pol and inhibits release^30^. The observation that virus release is increased in the presence of the PR D25N catalytic site mutation suggests that some premature activation of PR occurs even under wild-type conditions. To determine if the reduction of viral particles observed with the IN E152A or IN H12N mutants was a result of premature PR activation, we generated virions in the absence of presence of the PR inhibitor saquinavir (Fig. 4D). Indeed, particle release was fully restored for the E152A mutant in the presence of saquinavir, indicating that this mutation most likely promotes premature activation of the viral PR, reducing particle numbers but retaining processing of the FRET substrate in the virions that are released. In contrast, inhibition of PR by saquinavir did not fully restore release of particles for the IN H12N mutant (p < 0.0001), suggesting a more complex disruption of PR function.

### Patient-derived HIV viruses demonstrate considerable heterogeneity in protease activation that correlates with viral infectivity

**Figure 5:**
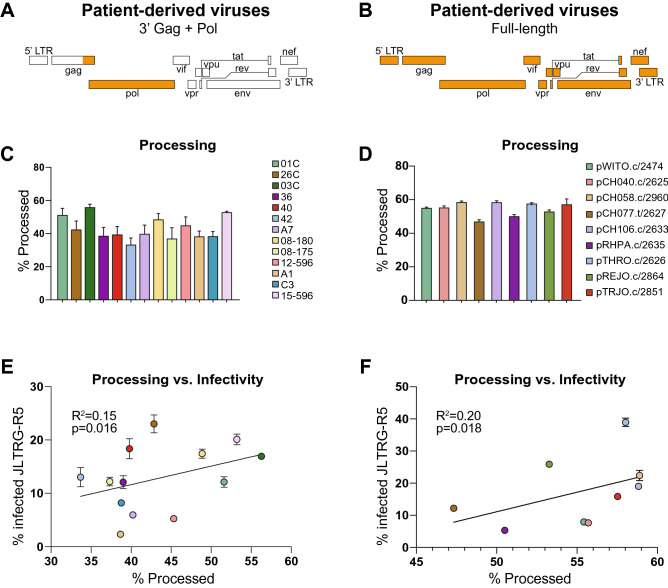
Patient-derived HIV viruses demonstrate heterogeneity in PR processing efficiency that contributes to viral infectivity. (**A, B**) Schematic of the 3′ Gag + Pol and full-length patient-derived viruses. Regions of the virus derived from patients are shown in orange; the NL4-3 backbone is shown in white. (**C,D**) Patient-derived viruses demonstrate a range of processing efficiencies ranging from 33.7–58.9%. (**E,F**) Processing of VIPER substrate correlated with infectivity of the 3′ Gag + Pol and full-length viruses on JLTRG-R5 reporter cells.

NFC is a transformative technology for monitoring the heterogeneity of viral particles due to its ability to rapidly interrogate millions of individual viruses. Having demonstrated that VIPER sensitively and accurately monitored viral PR activity, we next sought to determine whether there was patient-to-patient variability in PR activation levels using two panels of patient-derived viruses. The first panel was previously generated by purifying viral RNA from patient plasma and amplifying a ~ 3400 bp region consisting of the 3′ end of Gag and the entire Pol gene (3′Gag + Pol viruses), including protease, reverse transcriptase, and integrase. This patient-derived segment was cloned into a vector containing the HIV isolate NL4-3, resulting in a panel of viruses that vary in 3′Gag + Pol but are identical throughout the rest of the viral genome (Fig. 5A)^31,32^. A second panel of patient-derived viruses was obtained from the NIH AIDS Reagent Program and was previously isolated by single genome amplification (SGA) of plasma HIV RNA to obtain full-length, patient-derived viruses (Fig. 5B)^33,34^. These full-length viruses have mutations across the entire genome in all 16 HIV-encoded proteins and protein subunits, all of which have been implicated in viral infectivity and fitness.

To assess PR activation in patient-derived viruses, we co-transfected plasmids encoding VIPER and the 3′Gag + Pol or full-length patient-derived viruses into HEK293T cells. After 48 h, viruses were harvested and diluted to ensure single-particle analysis. Three to four independent preparations of each virus were made and analyzed for VIPER processing using NFC. We observed highly reproducible and substantial differences in PR activation among patient-derived viruses, ranging from 33.7 to 58.9% processing (Fig. 5C,D). Since PR activity is essential for viral replication, we next investigated whether the differences in PR processing between patient-derived viruses correlated with the infectivity of the virus. For these studies, we used the Jurkat LTR-GFP CCR5 + (JLTRG-R5) reporter cell line that expresses both HIV coreceptors—CCR5 and CXCR4—and produces GFP from the Tat-dependent viral LTR promoter upon infection with HIV. We observed a correlation between PR processing and infectivity in both the 3′Gag + Pol viruses (R^2^ = 0.15, p = 0.016) and the full-length viruses (R^2^ = 0.20, p = 0.018) (Fig. 5E,F). The low R^2^ values of these correlations are not unexpected and indicate that PR activation and processing efficiency is only one of multiple factors that collectively impact overall viral infectivity. These exciting findings demonstrate that patient-derived viruses vary substantially in PR activation—which has not been previously reported—and that these differences affect the infectivity of the viruses and may impact disease progression, particularly in untreated patients.

### VIPER accurately predicts drug resistance in patient-derived HIV viruses

**Figure 6:**
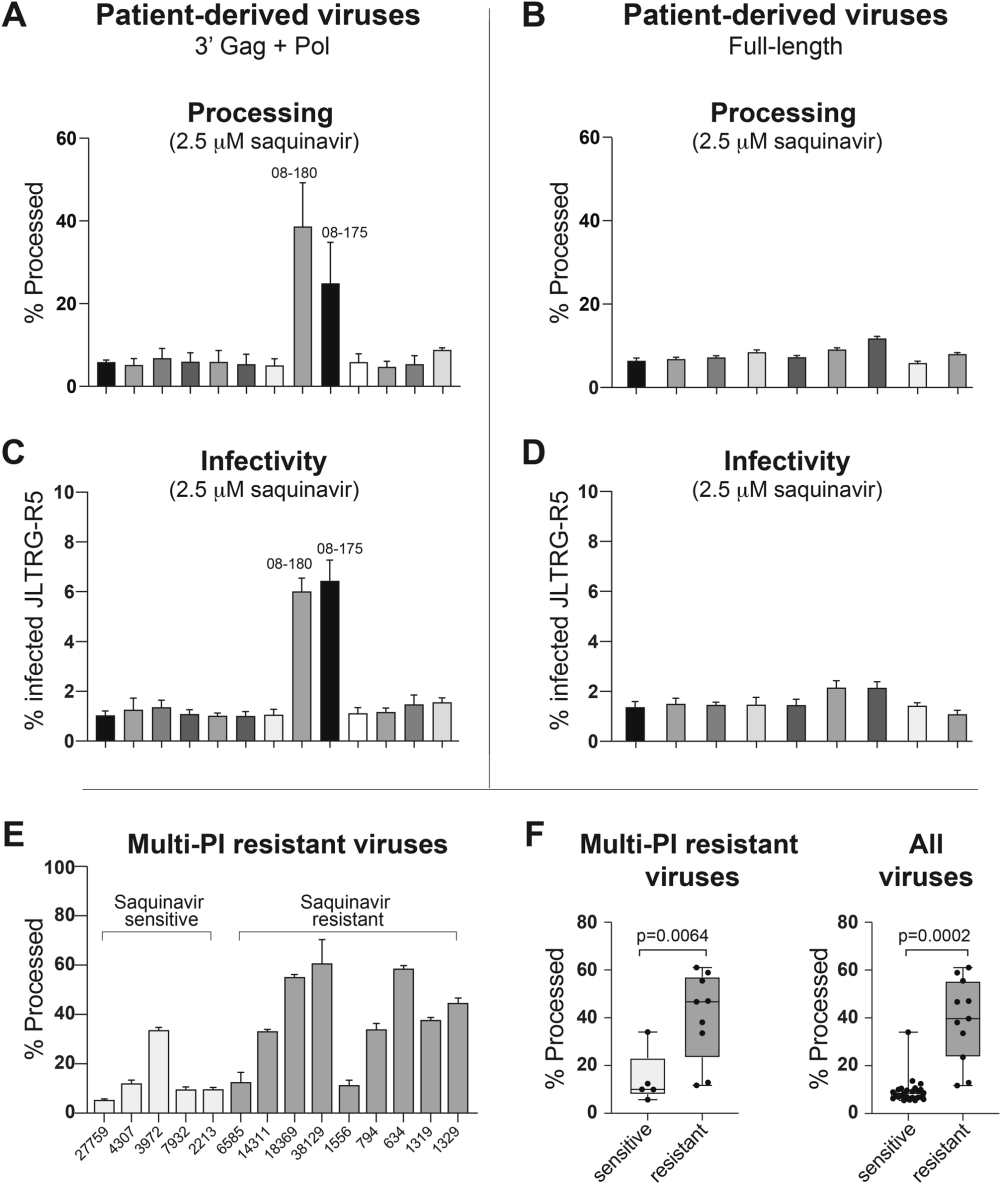
NFC accurately predicts drug resistance phenotype in patient viruses. (**A, B**) VIPER processing efficiency of patient-derived viruses produced in the presence of 2.5 μM saquinavir. Two 3′ Gag + Pol viruses, 08–180 and 08–175, demonstrated substantially elevated processing in the presence of saquinavir. These viruses were predicted to have intermediate- and high-level resistance to saquinavir, respectively, by sequence analysis. (**C, D**) Infectivity of patient-derived viruses on JLTRG-R5 cells in the presence of 2.5 μM saquinavir. The two patient-derived viruses demonstrating processing of VIPER in the presence of saquinavir also demonstrated elevated infection of target cells in the presence of drug. (**E**) VIPER processing efficiency was assessed in viruses generated from patient-derived, multi-protease inhibitor resistant proviruses. These variants have varying sensitivity to different PIs, with 5 viruses being classified as sensitive and 9 as resistant to saquinavir based on replication in the presence of drug^36^. NFC successfully identified 4/5 sensitive and 7/9 saquinavir-resistant viruses among this multi-PI resistant panel. (**F**) VIPER processing in the presence of saquinavir was significantly different between saquinavir-sensitive and -resistant viruses among the multi-PI resistant viruses (left panel) as well as among all patient-derived viruses (right panel) used in this study.

To assess the ability of NFC and VIPER to identify drug resistant HIV viruses, we also generated 3′Gag + Pol and full-length patient-derived viruses in the presence of the PI saquinavir (Fig. 6A,B). Two of the 3′Gag + Pol viruses (08-180 and 08-175) demonstrated substantial VIPER processing even in the presence of high-dose saquinavir. The remaining 3′Gag + Pol and all of the full-length patient viruses were inhibited by saquinavir. The 3′Gag + Pol viruses were originally cloned as part of a novel method to analyze drug resistance^35^ and had been sequenced and analyzed by the Stanford University HIV Drug Resistance Database. 3′Gag + Pol Virus 08-180 had seven major resistance mutations in PR: V32I, M46I, I47V, I54L, V82T, I84V, and L90M and five accessory mutations: K20T, L33F, K43T, G73S, and L89V and was predicted to have high-level resistance to all PIs. Virus 08–175 was found to have four major PI resistance mutations in PR: M46I, I54L, I84V, L90M and four accessory mutations: L10F, L33F, Q58E, and G73T and was predicted to have high-level resistance to all PIs except darunavir, to which it has intermediate resistance. The remaining 11 3′Gag + Pol viruses were all predicted to have viruses that are sensitive to PIs. The full-length viruses from the NIH AIDS reagent program were not analyzed for genotypic resistance but were derived from recently infected, antiretroviral-naïve patients who would be expected to have drug-sensitive viruses.

To determine if VIPER accurately predicted drug resistance of the full-length and 3′Gag + Pol patient-derived viruses, we also performed phenotypic analysis of resistance to saquinavir. For these experiments, viruses were produced in the presence of saquinavir and were then used to infect JLTRG-R5 cells, also in the presence of drug. Infections were performed with equal concentrations of virus after normalizing by viral RNA. As expected, the two 3′Gag + Pol patient-derived viruses with evidence of PI resistance using NFC—08–180 and 08–175—were able to infect JLTRG-R5 cells in the presence of saquinavir (Fig. 6C), providing functional confirmation of drug resistance in these viruses. All other 3′Gag + Pol and full-length patient viruses were inhibited by saquinavir (Fig. 6C,D). These data demonstrate that VIPER in combination with NFC successfully predicted the drug resistance phenotype for all 22 patient-derived viruses, highlighting the potential of this technology to rapidly and accurately assess viral characteristics critical to patient care. To assess the ability of VIPER to detect PI resistance under challenging conditions, we obtained a panel of multi PI-resistant patient-derived viruses with varying levels of sensitivity to saquinavir and other PIs. We generated viruses containing VIPER from these patient-derived viruses and assessed processing by NFC. As shown in Fig. 6E, 4 of the 5 multi-PI resistant viruses that were *sensitive* to saquinavir—as defined by replication in the presence or absence of drug using the PhenoSense assay^36^—also demonstrated low levels of VIPER processing in the presence of saquinavir. Conversely, 7 of the 9 multi-PI resistant viruses that were resistant to saquinavir showed elevated processing of VIPER in the presence of saquinavir. Of note, patient-derived virus 6585 was exactly at the cutoff for saquinavir resistance in the original study^36^ and did not infect JLTRG-R5 cells in the presence of saquinavir in experiments (data not shown); however, we are including it with the ‘saquinavir resistant’ viruses to maintain consistency with the original manuscript. Overall, the processing of VIPER was significantly different between saquinavir-sensitive and -resistant multi-PI resistant viruses (14.4 ± 11.2% v. 40.6 ± 18.1% processed viruses, p = 0.0064; Fig. 6F, left panel) and in all viruses used in this study (9.3 ± 5.6% v. 39.0 ± 17.2% processed viruses, p = 0.0002; Fig. 6F, right panel) and correctly predicted sensitivity to saquinavir in 33/36 (92%) of the patient-derived viruses used in this study.

## Discussion

Despite more than thirty years of investigation, many key questions about the activation of the HIV-1 protease (PR) remain unresolved, including how and when the precursor protease becomes activated during the assembly and budding process, the identification of host and viral factors outside of PR that contribute to its activity, and whether variability in PR exists among patient viruses that could contribute to clinical outcomes, particularly in untreated individuals. In this paper, we sought to overcome limitations of current assays for monitoring PR activity by leveraging the emerging field of nanoscale flow cytometry (NFC), which enables high-throughput analysis of individual submicron particles including exosomes, microvesicles, bacteria, and viruses. Despite extensive progress demonstrating heterogeneity in host protein incorporated into viruses^11^, the quantities and conformations of viral proteins^12–14^, size of particles^12,15^ and genome incorporation efficiency^16^, no previous NFC studies have attempted to monitor an enzymatic process such as HIV PR activity using this technology.

Here, we developed a novel FRET reporter called VIPER to study PR activity in individual viral particles using NFC. VIPER is selectively packaged into budding virions, undergoes sensitive and reproducible colorimetric changes in the presence of a functional viral PR, and correlates extremely well with Gag polyprotein processing. These results substantially advance the NFC field from a technical perspective by demonstrating that viral enzymatic processes and FRET constructs can be monitored even within the limited confines of a viral particle. In addition to these technical advances, our results break new ground for the clinical utility of the NFC technology. The current study is—to our knowledge—the first to use NFC to investigate patient-derived viruses and resulted in the novel observation of substantial inter-patient variability in PR activity that correlates with the infectivity of the viruses. Additionally, we demonstrate that VIPER processing by NFC was significantly different between viruses that were sensitive or resistant to the protease inhibitor saquinavir and that NFC accurately identified saquinavir resistance or sensitivity in 33/36 (92%) of patient-derived viruses tested. While these results are exciting, there are a number of limitations to the VIPER and NFC technology that make it unlikely to supplant sequencing-based drug resistance screening in the near future. First, the viruses tested in this study were chimeric viruses in which fragments of patient-derived HIV were inserted into a pNL4-3 HIV backbone. For efficient detection of drug resistance, the VIPER/NFC assay will need to monitor PR activity in intact, full-length patient isolates and without requiring extensive and costly cloning. Second, HIV exists in diverse quasispecies in patients and even low levels of pre-existing, drug-resistant viruses can rapidly lead to resistance and treatment failure. Therefore, clinical assays must be able to assess a large number of viral quasispecies within a patient to reliably detect low-frequency drug resistant viruses. While overcoming these challenges will require significant advances in the NFC field and VIPER platform, we believe the current study demonstrates that NFC is well suited to exploring viral heterogeneity in patients and its role in viral pathogenesis.

NFC analysis of HIV PR activity using VIPER has a number of significant advantages over existing assays. First, current assays predominantly study the activity of the mature viral PR and cannot assess the factors underlying the dimerization and autoprocessing of the precursor PR from the Gag-Pol polypeptide chains. In contrast, we find here VIPER is processed in HIV virions at the earliest stages of precursor PR dimerization and activation. Current PR inhibitors are far more effective at inhibiting the mature PR than the precursor PR still embedded in the polypeptide chain^26,37–40^. Combined with evidence that the precursor and mature PR have different dissociation rates^37, 41^, processing efficiencies^37^, and substrate preferences^42,43^, these results suggest that the two enzymes are conformationally distinct. The combination of NFC and VIPER provides the opportunity to screen for novel drugs affecting precursor PR dimerization and autoprocessing. Second, the combination of simultaneous monitoring of PR activity and quantitative viral release^17^ enables not only sensitive detection of mutations and drugs that affect these processes, but can also lead to rapid mechanistic insights. For instance, we found that the IN E152A mutation inhibited viral particle release but did not alter the processing of VIPER in the resulting particles. The restoration of particle release in the presence of saquinavir indicated that the IN E152A mutation results in premature activation of the viral PR and processing of Gag and Gag-Pol prior to the completion of assembly and budding, likely by facilitating dimerization of adjacent Gag-Pol polyproteins. Third, the robust quantification of the NFC assay in the context of intact viral particles allows for sensitive detection of slight variation in PR activity caused by mutations, drugs, or patient-to-patient heterogeneity. We believe this sensitivity enabled the novel observation reported here that differences in PR processing efficiency exist between patients and affect the infectivity of HIV viruses.

The correlation between patient heterogeneity in PR activation and viral infectivity has important implications for clinical care, particularly in the context of untreated patients or those failing antiretroviral therapy. Although viral fitness is clearly driven by other factors in addition to PR activity, a previous study has demonstrated slower disease progression in patients treated with protease inhibitors (PI) despite the emergence of PI-resistant viruses^44^. Combined with the results reported here, these findings demonstrate that mutations in PR—whether driven by drug selection, immune pressure, or other factors—can impact viral infectivity and the rate of disease progression in patients. We speculate that differences in PR activity due to viral sequences or host genetics and immune responses may contribute to different disease progression rates in untreated patients infected with differing subtypes of HIV-1.

In conclusion, assessing HIV-1 PR activity by NFC represents a powerful and significant advance over traditional methods of monitoring PR and here has revealed previously unreported heterogeneity in patient-derived viruses that affects viral infectivity. NFC is a transformative technology for exploring the ‘submicron world’ of exosomes, microvesicles, bacteria, and viruses that are increasingly appreciated to impact viral disease progression and host immunity. The ability to monitor enzymatic processes by NFC will significantly enhance the power of this technology to explore the biology and pathology of submicron particles in health and disease.

## Methods

### Plasmids and DNA

#### The NFC FRET-based viral protease reporter (VIPER)

The mUKG-mKOκ fluorescent pair was obtained from the Chicken Mermaid S118 plasmid, a gift from Vincent Pieribone (Addgene plasmid #53617; https://n2t.net/addgene:53617; RRID:Addgene_53617). The mUKG and mKOκ proteins were amplified independently by PCR and the HIV protease cleavage site SQNYPIVQ introduced via extended primers. The extended mUKG-SQNYPIVQ-mKOκ region was subsequently amplified by overlap PCR and introduced into the bla-Vpr plasmid (obtained from Robert Doms) in place of β-lactamase using Gibson assembly. Plasmids were confirmed by sequencing and purified using maxiprep kits (Qiagen). Control constructs containing mUKG-Vpr and mKOκ-Vpr alone were generated by amplifying the fluorescent genes by PCR and introducing them into the bla-Vpr plasmid by Gibson assembly.

#### HIV proviral cores

The NL4-3 ΔEnv EGFP Reporter Vector was obtained from the AIDS Reagent Program, Division of AIDS, NIAID, NIH, from Drs. Haili Zhang, Yan Zhou, and Robert Siliciano^45^. An HIV core lacking protease and Vpr, Gag CeFP Bgl-SL, was generously provided by Dr. Wei-Shau Hu at NCI, Frederick. This core was modified to remove the CeFP protein and was used to set gates for NFC.

#### 3′Gag + Pol HIV genomes

The viruses containing patient-derived 3′ Gag and entire Pol gene inserted into the NL4-3 proviral backbone were previously cloned during the development of VIRALARTS, an HIV phenotypic assay to simultaneously detect drug resistance to multiple classes of antiretroviral drugs^31^. Briefly, PCR products corresponding to the 3′ Gag (p2/p7/p1/p6)/PR/RT/IN-coding region of HIV were purified with a QIAquick PCR purification and introduced via yeast homologous recombination into the pRECnfl-TRPΔp2-INT-URA3 vector containing a near full-length HIV genome with the *Saccharomyces cerevisiae* uracil biosynthesis (URA3) gene replacing the 3′Gag(p2/p7/p1/p5)/PR/RT/IN HIV coding sequence.

#### Multi-protease inhibitor resistant proviruses

The Panel of Multi-Protease Inhibitor Resistant Infectious Molecular Clones was obtained through the NIH AIDS Reagent Program, Division of AIDS, NIAID, NIH from Dr Robert Shafer: Cat# 12464, pV20742-4^36^.

#### Full-length HIV genomes

The Panel of full-length transmitted/founder (T/F) HIV Infectious Molecular Clones was obtained through the AIDS Reagent Program, Division of AIDS, NIAID, NIH from Dr. John Kappes, (Cat. #11919). One virus from this panel, pSUMA.c/2821, reproducibly generated viruses with extremely low titers and was not included in the experiments.

#### Mutant HIV genomes

The HIV-1 NL4-3 Infectious Molecular Clone (pNL4-3) was obtained through the NIH AIDS Reagent Program, Division of AIDS, NIAID, NIH from Dr. Malcolm Martin (Cat# 114). PCR site-directed mutagenesis was used to modify the vector prior to assembly of the PCR products using NEBuilder HiFi DNA Assembly Master Mix (NEB). A D32N mutation was placed in p6 for the development of a mass spectrometry assay for protease activity in a separate manuscript and was confirmed to not alter the processing or infectivity of the virus (data not shown). The D32N mutant served as our wild type virus, which was subsequently used to generate the PR, IN, and cleavage site mutants. The entirety of Gag and Pol was confirmed by sequencing.

### Virus production and controls

All viruses were produced by co-transfection of an HIV core (as described in the Methods section under Plasmids and DNA) alone or with the VIPER plasmid in HEK293T cells. For nanoscale flow experiments, the cells were plated in a 6-well tissue culture dish at a density of 1.2 × 10^6^ cells per well, incubated overnight, and transfected with 3 μg of HIV core and 1.5 μg of the VIPER plasmid using PEI or calcium phosphate methods. For controls, cells were left untransfected to determine background from machine noise and extracellular vesicles (EVs) or transfected with the VIPER plasmid alone to assess uptake into EVs. All viral samples were fixed in 4% PFA prior to NFC analysis. For infection studies and western blots, HEK293T cells were plated into 10 cm dishes at a density of 4 × 10^6^ cells per plate. The following day, each plate was transfected with 9 μg HIV core and 5 μg VIPER if needed using PEI methods. 48 h post-transfection, supernatant was collected, filtered through a 0.45 μM filter, and concentrated using ultracentrifugation through a 20% sucrose cushion. The viral pellet was resuspended in PBS-/-. Virus was diluted and fixed in 4% PFA prior to NFC analysis. For assays involving saquinavir, the drug was added to plates at a concentration of 2.5 μM immediately following transfection.

### Nanoscale flow cytometry (NFC)

#### Biosafety

Analysis of infectious HIV particles by NFC poses potential safety risks to machine operators. In particular, flow cytometers that have sorting capabilities—such as the BD FACSAria used in these experiments—can potentially result in the production of aerosols that pose a biosafety hazard. For safety, all viruses were inactivated by 4% (PFA) prior to analysis by NFC. Furthermore, the FACSAria used in these experiments is contained in a negative-pressure BioBubble (Propel Labs) containment system equipped with a 0.3 μm HEPA filtration at 200 cfm airflow. The Case cytometry core routinely monitors system integrity using a “Glo-germ” protocol. All equipment and procedures are in compliance with the proposed NIH/International Society for the Advancement of Cytometry standards and approved by the Case Western Reserve University Environmental Health Safety office.

#### Analysis

NFC was performed using an 18-color FACSAria II SORP sorter (Becton Dickinson) equipped with 355 nm (60mW), 405 nm (100mW), 488 nm (100mW), 532 nm (150mW), 640 nm (100mW) lasers. The unprocessed FRET signal was detected using a 575/40 filter on the 488 nm laser while the processed mUKG signal was monitored using a 515/20 filter on the 488 nm laser. Viral particles were detected using the 575/26 filter on the 532 nm laser that stimulates mKOκ directly and generates a signal regardless of whether the reporter has been processed. Sheath fluid was pre-filtered at 0.2 μm pore for all experiments. All viruses were diluted into the single-particle range of the assay to avoid coincidental detection, as previously described^17^. At least 20,000 events were collected in triplicate or quadruplicate for all conditions.

#### Western blot

Viruses produced through transient transfection of HEK293T cells were resolved on a 4–15% gradient SDS gel (BioRad) and probed with either a polyclonal anti-Gag rabbit IgG antibody or with a polyclonal anti-HIV-1 protease antibody (NIH AIDS Reagent Program, Division of AIDS, NIAID, NIH: anti-HIV SF2 p24 polyclonal and anti-HIV-1 Protease polyclonal) and a secondary swine anti-rabbit HRP-conjugated antibody (Dako) at dilutions of 1:500 in 5% milk/0.1% PBS-Tween or using the iBind Western Device (Thermo Fisher). HRP activity was monitored using the Clarity ECL substrate (BioRad) and chemiluminescence imaged using an ImageQuant instrument (GE Healthcare Life Sciences) or the iBright Imaging System (Thermo Fisher). Band intensity was analyzed using ImageJ.

### Infectivity studies

Infection studies were performed using the T cell-based reporter cell line JLTRG-R5 (obtained through the NIH AIDS Reagent Program, Division of AIDS, NIAID, NIH: Jurkat LTR-GFP CCR5 + cells (JLTRG-R5) (Cat #11586), from Dr. Olaf Kutsch)^46,47^. Prior to infection, virus concentrations were normalized using the Lentivirus Rapid Quantitation Kit (Cell Biolabs). 1 × 10^5^ cells per well were plated into a 96 well flat-bottom plate and infected by incubating with virus for 48 h in the presence or absence of 2.5 μM saquinavir. 48 h post-infection, cells were stained with a live/dead fixable yellow viability dye (Invitrogen) and fixed in 1% PFA. Cells were analyzed for EGFP expression and viability using an LSRII flow cytometer (Becton Dickinson).

### Statistics

Statistical analyses were performed using two-sided Spearman correlation. All differences with a *p* value < 0.05 were considered statistically significant. Prism version 8.0.1 (GraphPad) was used for statistical analyses. P values are listed in the text or figure legends. In figures, p < 0.05 is denoted by a single asterisk (*), p < 0.01 by a double asterisk (**), and p < 0.001 by a triple asterisk (***).
